# Pivotal role of tissue-resident memory lymphocytes in the control of mucosal infections: can mucosal vaccination induce protective tissue-resident memory T and B cells?

**DOI:** 10.3389/fimmu.2023.1216402

**Published:** 2023-09-11

**Authors:** Stephanie Longet, Stephane Paul

**Affiliations:** ^1^ Centre International de Recherche en Infectiologie, Team Groupe sur l'immunité des muqueuses et agents pathogènes (GIMAP), Université Jean Monnet, Université Claude Bernard Lyon, Inserm, Saint-Etienne, France; ^2^ Centre d'investigation clinique (CIC) 1408 Inserm Vaccinology, University Hospital of Saint-Etienne, Saint-Etienne, France; ^3^ Immunology Department, iBiothera Reference Center, University Hospital of Saint-Etienne, Saint-Etienne, France

**Keywords:** mucosal immunity, tissue-resident memory immune responses, infectious diseases, protection, vaccination

## Introduction

Tissue-resident memory lymphocytes at mucosal surfaces have been shown to be critical in long-term protection following mucosal infection. Tissue-resident memory T cells (Trm) have been well characterized in animal models and in humans, while knowledge about tissue-resident memory B cells (Brm) is currently more limited. Due to specific features of tissue-resident memory lymphocytes such as cross-reactivity and polyfunctionality, Trm and Brm have been demonstrated to be an ideal target for vaccine strategies aiming to induce protection at mucosal surfaces. The route of vaccine administration, the choice of antigenic epitopes and the impact of microenvironment appeared to be crucial parameters in the development of vaccine-induced mucosal tissue-resident memory responses in animal models. However, it remains significant gaps in understanding systemic and local signals needed to establish and maintain protective mucosal Trm subsets without inducing pathogenic populations. In addition, the development of Brm is currently not well understood. Discovery of innovative recombinant antigens and identification of safe mucosal adjuvants will be crucial in the development of vaccine formulations efficient to induce Trm and Brm at mucosal surfaces. This Opinion article will describe current knowledge about mucosal Trm and Brm and vaccine approaches already tested to induce tissue-resident memory lymphocytes at mucosal surfaces. It will pinpoint gaps in knowledge. It will suggest research avenues and highlight considerations to design vaccine strategies inducing mucosal tissue-resident memory lymphocytes and it will provide suggestions to improve methodology to quantify mucosal tissue-resident memory responses.

## Characterization and features of Trm at mucosal surfaces

Three memory T cell subsets have been characterized: circulating effector memory T cells are abundant in non-lymphoid tissues, circulating central memory T cells are predominant in secondary lymphoid organs and non-circulating tissue-resident memory T cells (Trm) are able to persist in non-lymphoid tissues. Trm subset was discovered in parabiosis and tissue transplantation animal studies more than 15 years ago (1) and it is the most abundant memory T cell population. Trm have the ability to reside in mucosal tissues such as lung (2), nasal (3), gut (4), skin (5–8) and reproductive tract tissues (9), following infection (10). Trm were characterized in mice, non-human primates (NHP) (11) and humans (12–14). They were mostly defined by the high expression of the adhesion molecule CD69 which can be associated with an upregulation of the αE integrin CD103 (1), and by the downregulation of molecules preventing tissue exit such as CCR7 and CD62L (10). Other markers were associated with Trm such as CXCR3, CD49a (15) (16) or CD44 (1) in specific tissues. Regarding the functions of Trm, antigen-specific CD8 and CD4 Trm in the respiratory tract were shown to be associated with a better control of viral and bacterial infections (e.g. reduction of viral load (12), limitation of intracellular replication of bacteria (17)). In addition, several animal and human studies described the polyfunctionality of influenza- (18, 19) or respiratory syncytial virus (RSV)-specific CD8 Trm responses (20) as a key feature of protective Trm. The cross-reactivity of human Trm via recognition of conserved regions was also reported in the context of influenza (21, 22) and Severe Acute Respiratory Syndrome Coronavirus 2 (SARS-CoV-2) (3) infections. Another interesting feature of Trm is their potential innate-like functions helping in mucosal protection. It was demonstrated that CD8 Trm located in lung parenchyma of mice intranasally infected with influenza virus engineered to express ovalbumin as an antigen, could reduce the severity of a subsequent pulmonary bacterial infection through neutrophil recruitment (23). A similar bystander activation of T cells was observed in a Herpes Simplex Virus (HSV) murine challenge model. Indeed, it was shown that a subset of vaginal CD8 T cells which were unspecific to the HSV antigen used for immunization, could play a partial role in genital protection. In this HSV challenge model, both peripheral CD8 T cells able to migrate to inflamed vaginal tissue and vaginal CD8 Trm of irrelevant antigen specificity were involved in this innate-like function (24).

## Discovery of mucosal Brm

The development of tissue-resident memory B cells (Brm) following infection has been discovered in murine parabiosis (25), adoptive transfer (26) and depletion studies (27). The presence of lung Brm early post-infection was reported in mouse models of influenza (25) and pneumococcal pneumonia (28). It was shown that the induction of Brm required local encounter with antigen (25) and that Brm contributed to early plasmablast responses leading to the secretion of cross-neutralizing antibodies against viruses and bacteria (27, 28). Compared to Trm, Brm have been less characterized and specific markers have not been fully defined. However, it was shown that Brm expressed CD69, the hallmark of tissue-resident lymphocytes, in mice (25), NHP and humans (14). Other markers were also associated with Brm in mice such as CXCR3 and CD44 (25, 26, 28). In addition, antigen-specific CD73 positive and negative pulmonary Brm subsets were described in a mouse influenza model (25). In animal studies, the analysis of Brm is based on the discrimination of resident and circulatory B cells by intravenous labelling. In animal models and humans, gating strategies based on characterized memory B cell markers and similarities with Trm transcriptional profiles are also used to describe Brm subsets (29). Interestingly, a study found a potential intestinal Brm subset. Indeed, most CD19^+^CD27^+^ B cells in human intestine were CD45RB^+^CD69^+^ B cells. In addition, sets of gene expressed in lung Trm were enriched in this gut B cell subset suggesting that it could be an intestinal-resident population (30). The presence of Brm in other mucosal tissues such as skin or reproductive tract is currently unclear.

## Vaccine approaches to induce tissue-resident memory responses at mucosal sites

For more than two centuries, vaccination has been a successful global strategy to reduce the burden of several infectious diseases (31). Historically, the immunogenicity induced by vaccines was associated with systemic humoral response which can be easily measured in blood using antibody assays (32). However, for several decades, efforts have been put into the understanding of vaccine-induced cellular and local immune responses. Could the protective capacities of mucosal Trm and Brm be harnessed to improve immunity at mucosal barriers?

A range of vaccine approaches have especially been tested in mouse models to improve mucosal Trm development (33). The route of vaccine administration has been shown to play a crucial role. Growing evidences suggest that mucosal vaccines on their own or combined with systemic vaccines could be a promising strategy to enhance the development of mucosal Trm. For example, a study found that intranasal administration of live-attenuated influenza virus induced the development of CD4 and CD8 Trm in lungs, whereas systemic immunisation with live-attenuated influenza virus did not generate similar Trm response in mice (34). Similarly, intranasal immunization of mice with a chimpanzee adenoviral-based SARS-CoV-2 vaccine was shown to induce CD103^+^CD69^+^CD8 T cells in lungs, while vaccination by intramuscular route failed to generate pulmonary Trm cells (35). Another strategy named ‘prime and pull’ was tested to generate Trm in vaginal tract. Mice were subcutaneously immunized with an attenuated strain of HSV-2 (prime). Then, pro-inflammatory chemokines were applied to the vagina of mice in order to recruit HSV-specific CD8 T cells to this mucosal site (pull). Compared to the other experimental groups which were primed and boosted by intravaginal or subcutaneous routes only, the prime and pull strategy was the only one leading to the establishment of CD8 Trm in the genital mucosa. This study suggested that inflammation on its own could lead to the recruitment of Trm to genital mucosa and that a persistent antigen stimulation was not needed for the establishment of Trm (36). Similar findings were reported in nasal, upper respiratory tract (2) and skin (6). However, it was demonstrated that a local antigen encounter was needed to establish CD8 Trm in lungs (37–39). For instance, mice immunized by intraperitoneal route with influenza virus (prime) could exclusively generate pulmonary Trm following an intranasal immunization (pull) with CpG oligodeoxynucleotides combined with the antigen. The authors of this study suggested that circulating antigen-specific CD8 T cells could cause local tissue damage, which could play a role in Trm conversion (39). Interestingly, the importance of vaccine epitopes was pinpointed in a study describing the sequence design and immunogenicity of a CD8 T cell peptide Coronavirus Disease 19 (COVID-19) vaccine. This COVID-19 vaccine candidate was based on a range of 11 structural and non-structural SARS-CoV-2 proteins including conserved regions. The sequence was designed using SARS-CoV-2 immuno-dominant epitopes determined by screening and SARS-CoV-2 neoepitopes selected using a computational multi-neoepitopes based peptide vaccine approach, which had been shown to be safe and efficient in clinical trials evaluating a vaccine candidate against lung cancer. Following one subcutaneous vaccination in a mouse model, the COVID-19 vaccine candidate could induce a significant number of peripheral viral-specific CD8 T cells expressing Trm markers such as CD103 and CD49a, in spleen and draining lymph nodes. Even though the authors did not analyse Trm in lungs, this study highlights the importance of epitope selection to induce lymphocytes with a tissue-residency signature (40). Another study compared the functionality of T cells and Trm in lung biopsies collected for cancer suspicion in SARS-CoV-2 infected patients or individuals vaccinated with COVID-19 mRNA vaccines. The current spike-based COVID-19 mRNA vaccines were shown to induce similar SARS-CoV-2 spike-specific IFNγ CD4 T cell responses in lungs of vaccinees and convalescents, while antigen-specific CD8 T cell responses were not induced, neither in convalescents, nor in vaccinated individuals. Regarding tissue-resident memory responses in lungs, polyfunctional CD4 and CD8 Trm induction was shown to be limited post-vaccination compared to post-infection. A selection of SARS-CoV-2 epitopes, as previously described, could help to improve tissue-resident memory responses generated by the current COVID-19 mRNA vaccines (41). Determining the role of local antigen stimulation, mucosal inflammation and antigenic epitopes in specific vaccine strategies to induce tissue-resident memory lymphocytes is fundamental. These parameters may impact on the choice of the antigen, the administration route and the vector used to deliver the vaccine (10).

Regarding Brm, their induction has especially been evaluated in mouse pulmonary tissues (25). As it was determined that the establishment of pulmonary Brm post-infection required a local antigen encounter (25), it could be hypothesized that mucosal vaccination might also be beneficial to generate Brm in lung tissues at least. However, this hypothesis remains to be demonstrated. Interestingly, evidences revealed that the establishment of Brm pool did not correlate with the presence of Trm in the reproductive tract of female mice immunized with HSV (42). These data suggest that Trm and Brm responses could be induced in an independent manner at least in the reproductive tract. Trm and Brm development might have different kinetics and/or might require different local microenvironments. This correlation needs to be studied in other tissues given a lack of correlation might significantly impact on the development of vaccines targeting mucosal tissue-resident memory responses. Do specific vaccine approaches need to be developed to induce either Trm or Brm? It is currently unknown.

## Research avenues and considerations to design vaccines inducing protective tissue-resident memory lymphocytes at mucosal surfaces

### Understanding mucosal tissue-resident memory lymphocyte generation

A better understanding of Trm and Brm establishment and maintenance at mucosal surfaces is needed to tailor efficient vaccine strategies. Based on animal studies, two general models have been developed to explain Trm formation. The local divergence model suggests that Trm differentiate within tissues from pluripotent circulating effector T cells. The systemic divergence model proposes that there is a subset of circulating Trm precursors intended for migrating into tissues where they finish their differentiation into mature Trm (32). Regarding Brm formation, their origin has not been fully characterized and has been mainly based on lung Brm studies in mouse models. Influenza virus infection models suggest that Brm could originate from germinal centres in mediastinal lymph nodes or from germinal centre-like structures in the inducible bronchus-associated lymphoid tissues (25, 43). Identification of circulating Trm and Brm precursors by flow cytometry and transcriptomics using known tissue-resident memory markers and genes could be a way to find novel circulating lymphocyte populations sharing residency-promoting signature with Trm. This type of study could be performed before and after infection or vaccination in animal models. It would help to validate these models or hypotheses even though they might be non-exclusive (32), tissue- and context-dependent. Recently, some evidences have suggested the presence of precursor CD8^+^ Trm within circulation (44). In addition, some studies have demonstrated that Trm were able to egress and migrate to lymph nodes (45) or to distant mucosal sites (46). Understanding potential movements of tissue-resident memory lymphocytes can be crucial in order to optimize the routes of administration and to determine whether mucosal vaccination on its own or whether mucosal vaccination after a systemic prime is the best strategy. The influence of the priming route should be studied using parabiosis mouse models.

Some studies suggested that specific cytokines or metabolites could impact on Trm formation. Indeed the role of TGFβ has been described in the generation of lung (47), nasal/upper respiratory tract (2), gut (48) and skin (7, 49) CD103^+^ Trm. Modulation of TGFβ might be a key parameter to optimize vaccine strategies aiming to induce Trm including lung CD8 Trm (50, 51). A role of other cytokines such as IL-10 (52), IL-21 (53), IL-15 (54, 55) and IL-1/IL-2 (56) was also described in Trm formation in lungs or other tissues. Recently, it has been demonstrated that a prime of T cells in the mesenteric lymph node of mice infected with *Listeria monocytogenes* by oral route, licensed T cells to differentiate into CD103^+^ T cells in intestine and that this licensing was regulated by retinoic acid (57). Systemic and mucosal signals required to generate tissue-resident memory lymphocytes need to be further elucidated in order to design vaccine formulations leading to an appropriate microenvironment. The use of knockout mice for specific cytokines or their receptors, mouse Cre-LoxP system, as well as reporter systems may help to understand the role of systemic and mucosal signalling pathways and microenvironments involved in the expansion and responsiveness of tissue-resident memory lymphocytes post-vaccination at different mucosal sites. Gene-based systems such as transcription factor profiling of historical activity in specific tissues (58) or novel DNA-based memory system (59) could help to define the epigenetic state of potential precursor populations but also early signals linked to tissue-resident memory lymphocyte formation.

Complicating the picture of tissue-resident memory responses, sub-populations of CD4 Trm have been described based on specific cytokine profiles. Indeed, lung Trm1, Trm2, Trh, Trm17 have been characterized following different respiratory infections (53) (60). However, it is unclear whether their differentiation requires different signalling pathways, specific microenvironments and whether their persistence is similar in mucosal tissues. It is also important to consider that some particular Trm subsets have been associated with persistent immunopathology after viral infection or with chronic diseases (60). For instance, an expansion and activation of Trm17 was identified in bronchoalveolar lavage fluid from severe COVID-19 patients following SARS-CoV-2 clearance. The characterization of this subset showed a pathogenic cytokine profile associated with severe disease and lung damage (61). It was also observed that an enrichment of CD69^+^CD103^-^ Trm population in bronchoalveolar lavage fluid collected from patients with post-COVID-19 acute sequelae negatively correlated with their lung function (62). A specific CD103^+^CD161^+^CCR5^+^CD4^+^Trm sub-population was also reported to be predominant in the intestine of Crohn’s disease patients (63). These examples of pathogenic Trm profiles pinpoint the importance to clearly define the parameters leading to the establishment of protective tissue-resident memory lymphocytes at mucosal surfaces following vaccination. The development of protective or exuberant tissue-resident memory lymphocytes might be related to the type of stimulus, the persistence of stimulation, the local environment and the type of activated signalling pathways. Qualitative and quantitative differences between protective and pathological tissue-resident memory responses need to be elucidated. During the development of vaccine candidates aiming to induce mucosal Trm or Brm, it will be crucial to determine the cytokine profile of tissue-resident memory lymphocytes generated at mucosal surfaces after vaccination in order to evaluate the maintenance of mucosal homeostasis even though limited inflammation can transiently be induced by vaccination. Spatial transcriptomics *in situ* may be a critical approach to detect inflammation associated with tissue-resident memory lymphocyte populations (64).

### Optimizing vaccine formulations

Given the ability of tissue-resident memory lymphocytes to generate cross-reactive immune responses specific to conserved epitopes, designing recombinant antigens which include conserved epitopes might be of great interest. Systems vaccinology and artificial intelligence could be approaches which should be explored to predict epitopes able to induce tissue-resident memory responses. In addition, the role of adjuvants may be essential to enhance and tailor tissue-resident memory responses at mucosal sites. Some promising adjuvants administered by mucosal route have been already identified in preclinical models. Marinaik et al. showed that acrylic-acid-based adjuvant associated with a Toll-like receptor agonist glucopyranosyl lipid adjuvant was the most effective vaccine formulation to induce influenza-specific CD103^+^ CD8 Trm in lungs of mice immunized by intranasal route (65). Using the same administration route, it was also shown that influenza antigens associated with IL-1β enhanced the number of antigen-specific CD103^+^CD69^+^ Trm in lungs of mice (66). Interestingly, some adjuvants administered by systemic route have shown to be able to enhance the induction of mucosal cellular responses. Indeed, it was reported that all-*trans*-retinoic acid administered by intraperitoneal route could enhance the frequency of antigen-specific memory T cells in murine intestine (67). Woodworth et al. demonstrated that CAF^®^10b, a liposomal adjuvant administered by intramuscular route, could prime T cells in order to recall them in the lungs or skin using the antigen only administered by intratracheal and intradermal routes in NHP (68). If all-*trans*-retinoic acid or CAF^®^10b adjuvants are beneficial to induce mucosal tissue-resident memory responses, it remains to be confirmed. The main challenge to design vaccine formulations able to induce mucosal responses including mucosal Trm/Brm is the current lack of adjuvants licensed for mucosal administration in humans (69). Identifying effective and safe mucosal adjuvants is a key factor to pursue the development of vaccine strategies to generate mucosal tissue-resident memory responses. Unfortunately, the lack of *in vitro* predictive assays for adjuvants does not help and *in vivo* models remain the gold standard for these analyses (70).

Finally, evidences have shown that sex (71) and age (60) (72) could impact on tissue-resident memory response profile and functionality. These parameters need to be further evaluated in the context of vaccine development. Vaccine strategies used to generate Trm/Brm should be tested in both female and male animals, as well as aged animals at some stages of development. It will help to tailor vaccine strategies aiming to induce protective Trm and Brm responses in human populations with different pre-existing chronic mucosal conditions. In addition, the role of microbiota or microbiota-derived metabolites in the development, maintenance, metabolism and modulation of tissue-resident memory lymphocytes also have to be considered (73) especially in the context of mucosal vaccination.

### Evaluating tissue-resident memory responses

The gold standard to analyze mucosal Trm and Brm responses remains animal models and the type of animal models is a crucial parameter. Inbred mice are commonly used in the studies. However, they may not be the best models to analyze tissue-resident memory responses at mucosal surfaces. Even though it remains challenging to mimic multiple mucosal exposures to a range of pathogens impacting on polyfunctional and polyreactive Trm and Brm in animal models, the use of outbred mice could better recapitulate observations found in humans as developing more tissue-resident memory lymphocytes in non-lymphoid tissues (74).

To evaluate the efficacy of vaccine strategies able to enhance Trm and Brm responses in humans, robust sampling and quantification methods are required to analyze mucosal tissue-resident memory responses. Analysis of post-mortem tissues or tissues after resection surgery is currently the best way to study tissue-resident memory responses at mucosal surfaces by flow cytometry or histological staining (75). Following SARS-CoV-2 infection, human nasal tissue-resident memory T cells have been recently analyzed using specific device for nasal sampling (76). However, isolation of human Trm and Brm from mucosal surfaces remains challenging. Consequently, defining correlations between peripheral markers and mucosal tissue-resident memory responses is crucial to include the analysis of mucosal tissue-resident memory responses in human vaccine trials. Peripheral markers could be based on mucosal homing markers expressed on circulating lymphocyte populations (77) or the circulation of tissue-resident memory precursors.

## Conclusion

**Figure 1:**
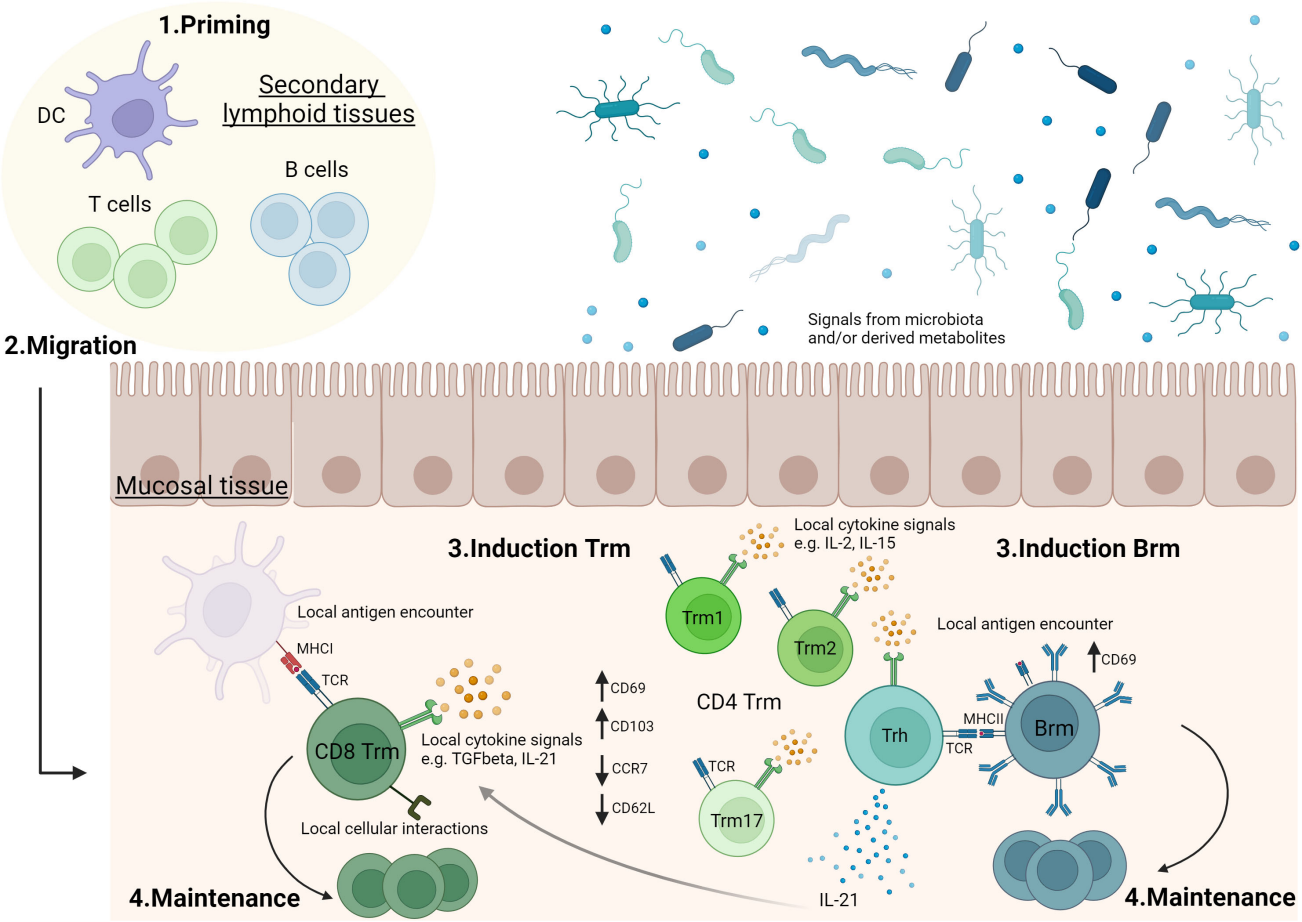
Generation of tissue-resident memory lymphocytes in mucosal tissues.1. T and B cells are activated in secondary lymphoid organs (e.g. lymph nodes, mucosa-associated lymphoid tissues). 2. Following activation, lymphocytes can migrate to mucosal tissues where they will convert into tissue-resident memory T cells (Trm) and B cells (Brm). 3 & 4. In mucosal tissue, antigen stimulation, cytokine signals and/or cellular interactions can drive mucosal tissue-resident memory lymphocyte induction and play a role in their maintenance. The role of these parameters may differ according to the type of mucosal tissue and there are still significant gaps in the current knowledge. For example, the role of local antigen encounter (37–39), TGFβ (50, 51), IL-2 (56), IL-15 (54) or IL-21 (53) in Trm induction, as well as the formation of sub-populations of CD4 Trm have been described in lungs (53, 60). Evidences showing a role of microbiota or microbiota-derived metabolites in Trm modulation have been reported, especially in intestine (73). Vaccination may impact on priming, migration, generation and maintenance of Trm and Brm in mucosal tissues. Trm1: tissue-resident memory CD4 T cells secreting Th1 cytokines. Trm2: tissue-resident memory CD4 T cells secreting Th2 cytokines. Trm17: tissue-resident memory CD4 T cells secreting Th17 cytokines. Trh: tissue-resident memory CD4 T helper cells. The figure was created with BioRender.com.

**Figure 2:**
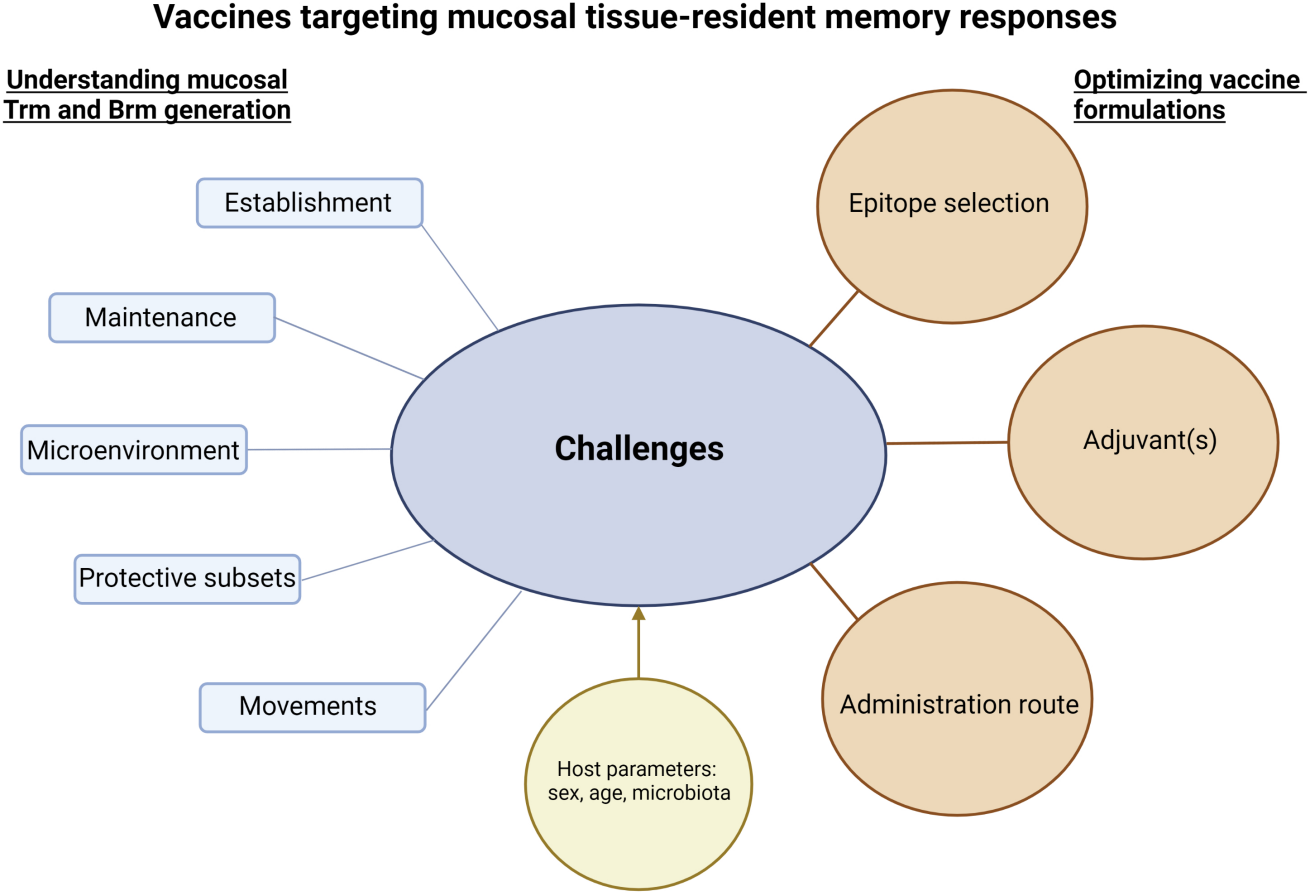
Challenges and considerations to develop vaccines targeting mucosal tissue-resident memory responses. Successful vaccine strategies to induce mucosal tissue-resident memory responses will be based on a better knowledge of the establishment and maintenance of protective Trm and Brm in mucosal tissues. In addition, understanding their potential movements to specific mucosal tissues and/or the movements of their precursors, as well as microenvironments needed to lead to functional Trm and Brm will be crucial. Interactions between Trm and Brm responses and host parameters such as sex, age or microbiota should be assessed. The sum of this knowledge will be essential to optimize vaccine formulations. The figure was created with BioRender.com.

Vaccines able to induce long-term mucosal responses are needed to improve protection against infection at mucosal surfaces. Expanding polyfunctional and cross-reactive tissue-resident memory responses using mucosal vaccination on its own or combined to systemic vaccination looks a promising way to reach this goal. An ideal vaccine would induce controlled and balanced Trm and/or Brm responses at mucosal sites (**Figure 1**). For this purpose, a better knowledge is needed to understand the formation of effective tissue-resident memory responses at mucosal surfaces and to determine the specific environment needed in each mucosal tissue to generate protective Trm and Brm subsets. Identifying effective mucosal adjuvants able to induce mucosal tissue-resident memory responses is a key parameter to optimize vaccine formulations (**Figure 2**). However, it will be challenging to move vaccine candidates into clinical trials if there are not any standard procedures to quantify Trm and Brm responses in human mucosal tissues. Identification of peripheral markers correlating with Trm and/or Brm responses could be an easy way to evaluate mucosal tissue-resident memory responses post-vaccination in humans.

## Author contributions

SP and SL have written the manuscript. All authors contributedto the article and approved the submitted version.
